# Associations Between Cognitive Function, Depression, and Olfactory Function in Elderly People With Dementia in Korea

**DOI:** 10.3389/fnagi.2021.799897

**Published:** 2022-01-11

**Authors:** Hyegyeong Cha, Sisook Kim, Yedong Son

**Affiliations:** ^1^Department of Nursing, Namseoul University, Cheonan-si, South Korea; ^2^College of Nursing, Woosuk University, Wanju-gun, South Korea

**Keywords:** dementia, elderly, depression, olfactory function, cognitive function

## Abstract

Early detection is important for delaying or preventing cognitive impairment. Since olfactory dysfunction and depression are common symptoms of cognitive dysfunction, they may serve as measurable risk indicators. This study was designed to identify the relationship between olfaction, depression, and each domain of cognitive function in elderly dementia patients in South Korea. Study participants were 108 patients who visited the outpatient clinic between March and September 2019. More significant impairment of olfactory function was found in those with mild (7.48 ± 1.28) or moderate (7.37 ± 2.22) test scores of the Expanded Clinical Dementia Rating (CDR) scale than in those with questionable scores (20.58 ± 6.18). The language domain of cognitive function, age, and education level showed 39.2% explanatory power for olfactory function (*F* = 5.591, *p* < 0.001). It is expected that assessment of olfactory function in elderly people can lead to the early detection, diagnosis, and treatment of dementia. Furthermore, it is important for future studies to confirm the relationship between each domain of cognitive function and olfactory function according to the type of dementia and to establish criteria for screening dementia in order to utilize olfactory function as a clinical marker.

## Introduction

South Korea is currently undergoing rapid population aging (Statistics Korea, 2021), leading to a steep increase in the number of elderly people with dementia. Among people aged 65 and older, 4.8 and 11.2% were reported to have dementia in 2010 and 2019, respectively (National Institute of Dementia, 2021). Thus, early detection of dementia in elderly people and the development of interventions are major priorities.

Dementia is a progressive disease characterized by the impairment of two or more cognitive domains, which causes difficulties maintaining social, occupational, and self-care activities (Arvanitakis et al., 2019). Dementia is primarily associated with deficits in the memory domain, along with deterioration of the language, visuospatial, and executive domains (Arvanitakis et al., 2019). Depending on the type of dementia, the affected cognitive domains are different. Alzheimer’s disease, for example, is mainly accompanied by loss of short-term memory, whereas vascular dementia leads to a decline in attention and executive function impairment and Lewy body dementia leads to functional deficits in visuospatial processing and attention (Seo, 2018). A decline in memory function is associated with AD, whereas a decline in attention function is associated with vascular dementia and a decline in visuospatial function is associated with Lewy body dementia. As such, the degree of deterioration of specific domains of cognitive function in people with dementia depends on the causative disease of dementia. Therefore, dementia is diagnosed and evaluated according to the cause of dementia and an assessment of cognitive domains.

Decreased olfactory function is a common phenomenon that accompanies chronological aging in the elderly. In a study of the elderly in Korea, 80.3% of participants were found to have olfactory function impairment (Seo et al., 2009). Olfactory dysfunction is accompanied by gustatory dysfunction, which can lead to loss of appetite and weight (Lee et al., 2007). Severe olfactory dysfunction has a particularly strong negative influence on quality of life and daily living behaviors (Seo et al., 2009; Yuan and Slotnick, 2014). However, although decreased olfactory function causes various problems in elderly people, its significance has been overlooked compared to vision and hearing (Seo et al., 2009).

It is known that olfactory function is also related to cognitive function (Lee et al., 2007; Domellöf et al., 2017; Marin et al., 2018). In a study examining olfactory function in patients with Parkinson’s disease, 73% of patients also had olfactory dysfunction, and the risk of dementia was 3.1 times higher among those with olfactory dysfunction than among those without it (Domellöf et al., 2017). As such, decreased olfactory function has been reported as an early symptom of Parkinson’s disease and Alzheimer’s disease (Marin et al., 2018). Therefore, it is believed that olfactory dysfunction can serve as an important clinical marker for early prediction and diagnosis of degenerative brain diseases such as dementia (Fullard et al., 2017; Marin et al., 2018). In addition, although olfactory function is known to be related to the executive and memory domains of cognitive function (Hedner et al., 2010). Lower language ability was reported to be associated with lower olfactory function (Larsson et al., 2005; Turana et al., 2020), and it was found that impaired olfactory function often accompanied language impairment (Tonacci and Billeci, 2018). However, there is a paucity of research on the relationship between olfactory function and the specific cognitive domains in dementia patients with cognitive decline.

Depression is also common in patients with dementia (Taylor, 2014). Prolonged depression can be a risk factor for dementia, and depression can be a prodrome of dementia or a consequence of prolonged dementia (Bennett and Thomas, 2014). White matter changes due to pathophysiological changes have been observed in the brains of patients with depression or dementia (Korczyn and Halperin, 2009). Depression and dementia can have various influences on individuals and society by not only lowering the quality of life of patients and their families, but also increasing mortality among elderly people as well as the overall socioeconomic burden (Bennett and Thomas, 2014).

Depression has also been found to be associated with olfactory function. Olfactory function has an influence on emotions (Yuan and Slotnick, 2014); studies on patients with depression found that olfactory function was significantly impaired in people with depression (Pause et al., 2001; Seo et al., 2009). Accordingly, it has been suggested that olfactory function tests could be used as a complementary marker for evaluating the effectiveness of treatments for depression as well as dementia (Marine and Boriana, 2014). However, according to a systematic review of the literature on the relationship between depression and olfactory function, while some studies observed a significant difference in olfactory function between groups with and without depression, the difference was not significant in other studies (Taalman et al., 2017), which underscores the need for further research on the relationship between depression and olfactory function. In particular, people with depressive symptoms are at a higher risk of dementia (Richard et al., 2013). Therefore, it will be helpful to clarify the risk of dementia by investigating whether there is a difference in olfactory function between dementia patients with depression and without depression.

Therefore, this study aimed to examine the influence of each domain of cognitive function and depression on olfactory function among elderly people with dementia in order to facilitate early detection of depression among elderly people with dementia and provide basic data for using the association between olfactory function and each domain of cognitive function to differentiate between causes of dementia.

This study investigated the effects of specific cognitive function domains and depression on olfactory function in elderly people with dementia. The specific objectives were as follows:

1) To identify participants’ general characteristics and differences in olfactory function.
2) To assess participants’ olfactory function.
3) To identify differences in olfactory function according to depression.
4) To identify differences in olfactory function according to cognitive function.
5) To identify factors influencing olfactory function.

## Materials and Methods

### Study Design

This descriptive research study was designed to examine the relationship between olfactory function, depression, and the various domains of cognitive function (attention, language, visuospatial, memory, and frontal/executive) in elderly people with dementia.

### Study Participants

The participants of this study were elderly people aged 65 or older with cognitive impairment. Patients who received dementia testing due to suspected dementia in Cheonan, South Korea were considered the target population. This study included a convenience sample of 127 patients who visited Cheonan Medical Center and met the inclusion criteria. Using the Expanded Clinical Dementia Rating (CDR) tool (Choi et al., 2001), which measures the degree of overall cognitive function of dementia patients, elderly people with dementia (CDR scores of 0.5 to 2.0, in which communication is possible) were enrolled as participants in this study. Patients were excluded from the study if they had nasal or sinus problems that could affect the examination, took drugs that can cause gustatory dysfunction, such as antirheumatic drugs or anticancer drugs, or had a history of disease that could affect olfaction and cognitive function, such as brain injury.

The number of participants was determined using G^∗^Power3.1.9.2, a program for statistical power analysis (Cohen, 1988). In the regression analysis, when the large effect size was 0.35, the significance level was α = 0.05, power was (1-β) = 0.95, number of predictors were 10, the total number of participants required was 80.

### Research Tools

The general characteristics of participants included in the analysis were sex, age, marital status, education level, religion, household type, prescription medication, CDR scores, and the degree of participants’ subjective perception of olfaction was included in the analysis to encompass olfaction-related characteristics.

#### Cognitive Function Evaluation Tool

The Seoul Neuropsychological Screening Battery II (SNSB II) (Kang et al., 2012) was used for a detailed evaluation of the participants’ cognitive function. The SNSB II is composed of five domains: attention, language, visuospatial, memory, and frontal/executive. The SNSB II includes various subtests that evaluate each domain of cognitive function and provides corrected scores considering age, sex, and education level. Among diagnostic tests for dementia, the SNSB II has a high interrater reliability (i.e., reliability between raters) and high sensitivity for making comparisons according to cognitive function domain and evaluating initial cognitive decline, with the results being expressed as a percentage. In a study by Lee et al. (2019), in order to confirm the interrater reliability, Kendall’s coefficient (W) was used to confirm whether the SNSB II evaluation scores of patients were consistent among medical staff. Cohen’s kappa was used in comparisons of the SNSB II evaluation score for each medical staff member and the existing dementia diagnostic test. As a result, the interrater reliability was high, with W = 0.86 (*p* < 0.0.01) and kappa = 0.84–1.0. In the current study, the test was performed by a trained researcher and results were evaluated by a neurologist. Then, the percentile was calculated for each cognitive domain using the standard score, meaning that, for each domain, participants with a score of less than 16 were considered to have cognitive impairment.

#### Olfactory Function Evaluation Tool

Olfactory function was evaluated using the University of Pennsylvania Smell Identification Test (UPSIT) (Doty et al., 1984). The UPSIT is a test in which testers or participants themselves scratch 40 different odor patches and select correct answers among four options. The total score is calculated as the sum of the scores for correct answers: 0–5 points indicate probable malingering, 6–18 points indicate total anosmia, 19–25 points indicate severe microsmia, 26–29 points indicate moderate microsmia, 30–33 points indicate mild microsmia, and 34–40 points indicate normosmia. At the time of the development of the tool, test-retest reliability using the Spearman-Brown formula was high (*r* = 0.918). In the current study, the researchers themselves scratched odor patches and then instructed the participants to smell them. For participants who could not read, the researchers read the four options to the participants aloud and recorded their answers. Those who could read recorded their own answers.

#### Depression Assessment Tool

Depression in participants was assessed using the tool designed by Cho et al. (1999). This tool is a standardized, short, Korean-language version of the Geriatric Depression Scale (SGDS), which was originally developed by Yesavage et al. (1983) and abridged by Sheikh and Yesavage (1986). The tool is a 15-item self-reported depression scale. Since each has a “yes” or “no” response, it is particularly useful for screening and assessing depression in elderly people. In light of the cognitive impairment of the study participants, the researchers read the questions to participants aloud and the participants answered verbally. Negative questions were scored inversely. This study used a cutoff of 8 or more out of 15 points to define depression, in accordance with Cho et al. (1999). In the study by Cho et al. (1999), Cronbach’s α for reliability was 0.886, and in the current study, it was α = 0.918.

### Data Collection

The study was conducted after obtaining approval from the institutional review board of the researchers’ institution (IRB No. 1041479-HR-20190-002), and data collection was initiated after receiving official written approval for the collection of patient data from Cheonan Medical Center between March 2019 and September 2019. Those who visited Cheonan Medical Center and were diagnosed with dementia were administered questionnaires and given examinations after signed consent forms were obtained from patients who voluntarily indicated their intention to participate in the study after the study purpose and goal were explained to them in person by researchers. The amount of time required for evaluating general characteristics and olfactory function was approximately 30 minutes per participant. For administering the SNSB II and SGDS, we sought cooperation from the hospital to obtain patient data. Of the total 127 participants, 19 were excluded from the analysis due to unsuitability for the study purpose, failure to answer questions, or lack of participation in tests during research. Thus, data on 108 participants were used for the statistical analysis.

### Data Analysis

The collected data were analyzed using the SPSS 21.0 statistical program. The general characteristics of the participants were calculated as numbers and percentages or means and standard deviations. Differences in olfactory function according to general characteristics were analyzed using the *t*-test and analysis of variance, and *post hoc* analysis was performed using the Scheffé test. To examine the relationship between depression and cognitive function, the trend of categorical data was analyzed using linear association, and the analysis of covariance (ANCOVA) was used to analyze differences in olfactory function according to depression and cognitive function. Factors affecting olfactory function were analyzed using hierarchical regression. Autocorrelation of the errors was tested using the Durbin-Watson statistic, and it was confirmed that there was no multicollinearity problem based on the tolerance and variation inflation factor values. Residual analysis was performed to confirm the linearity of the model and the assumption of normality of the error term and equal variance.

## Results

### General Characteristics of Participants and Differences in University of Pennsylvania Smell Identification Test Scores

In total, 53 participants were men (49.1%) and 55 were women (50.9%), with an average age of 75.74 ± 6.63 years. Forty-nine (45.4%) were aged 75 or older, 69 (27.8%) were unschooled or had an elementary school-level education, and the majority of participants were not religious (57 patients, 52.8%). Most of the participants took prescription medication (102 patients, 94.4%), most commonly for blood pressure (65 patients, 63.7%), followed by diabetes (34 patients, 33.3%) and stroke (31 patients, 22.5%). Most participants were non-smokers (98 patients, 90.7%), while only 10 participants (9.3%) were smokers.

There were significant differences in the integrity of olfactory function according to age, education level, household type, and overall cognitive function. Olfactory function scores were higher for participants under 75 years of age (19.39 ± 7.11) than for those who were 75 or older (14.90 ± 8.76), and scores were significantly higher for those who had a college-level education (23.75 ± 6.52) than for those who were unschooled (12.73 ± 9.61). Scores were higher for those living with spouses (18.83 ± 8.33) than for those living with spouses and other family members (12.27 ± 8.07). Those with questionable CDR scores had higher olfactory function scores (20.58 ± 6.18) than those with mild or moderate CDR scores. There was, however, no difference in olfactory function according to sex, marital status, religion, prescription medication and smoking (Table 1).

**TABLE 1:** General characteristics of participants and differences in UPSIT scores (*N* = 108).

Characteristics	Categories	*n* (%)	UPSIT	t or F (*p*)
Sex	Men	53 (49.1)	17.92 ± 7.64	1.215 (0.227)
	Women	55 (50.9)	15.98 ± 8.90	
Age (max, min)	75.74 ± 6.63 (65, 95)			
	< 75	49 (45.4)	19.39 ± 7.11	2.884 (0.005)
	≧75	59 (54.6)	14.90 ± 8.76	
Marital status	Married	63 (58.3)	17.87 ± 8.47	1.391 (0.167)
	Unmarried (Widowed/Separated/Divorced)	45 (41.7)	15.62 ± 8.02	
Education level	None^a^	26 (24.1)	12.73 ± 9.61	4.129 (0.004)
	Elementary school^b^	43 (39.8)	16.47 ± 7.01	a < e^†^
	Middle school^c^	15 (13.9)	19.07 ± 8.20	
	High school^d^	16 (14.8)	19.63 ± 7.20	
	College and over^e^	8 (7.4)	23.75 ± 6.52	
Religion	Yes	51 (48.2)	16.94 ± 8.38	−0.007 (0.994)
	No	57 (52.8)	16.93 ± 8.34	
Household type	Single household^a^	33 (30.6)	17.00 ± 7.43	5.204 (0.007)
	Spouse only^b^	53 (49.1)	18.83 ± 8.33	b > c^†^
	Spouse and other family^c^	22 (20.4)	12.27 ± 8.07	
Prescription medication	Yes	102 (94.4)	17.11 ± 8.29	0.888 (0.377)
	Hypertension	65 (63.7)		
	Diabetes mellitus	34 (33.3)		
	Cerebro-cardiovascular disease	31 (30.4)		
	Other	23 (22.5)		
	No	6 (5.6)	14.00 ± 9.23	
Smoking	Yes	10 (9.3)	17.37 ± 8.37	1.704 (0.091)
	No	98 (90.7)	12.70 ± 6.83	
CDR scores	Questionable^a^	63 (58.3)	20.59 ± 6.18	29.349 (<0.001)
	Mild^b^	34 (31.5)	7.48 ± 1.28	a > b,c^†^
	Moderate^c^	11 (10.2)	7.37 ± 2.22	

*^†^Statistical analysis method.*

### University of Pennsylvania Smell Identification Test Scores of Participants

Scores for olfactory function were classified into five categories: probable malingering (false positive), total anosmia (complete olfactory loss), severe anosmia (severe olfactory loss), moderate microsmia (moderate olfactory loss), and mild microsmia (mild olfactory loss). Total anosmia (37 patients, 35.2%) and severe anosmia (38 patients, 35.2%) were most common among the participants (Table 2).

**TABLE 2:** UPSIT scores of participants (*N* = 108).

UPSIT^1^	Scores	*n*	%
Probable malingering	0–5	14	13.0
Total anosmia	6–18	38	35.2
Severe anosmia	19–25	38	35.2
Moderate microsmia	26–29	15	13.9
Mild microsmia	30–33	3	2.8

*^1^UPSIT, University of Pennsylvania Smell Identification Test.*

### Differences in Olfactory Function According to Depression

Olfactory function was lower in the participants with depression (14.48 ± 8.00) than in the participants without depression (17.92 ± 8.30), but there was no statistically significant difference (*F* = 0.510, *p* = 0.477) (Table 3).

**TABLE 3:** Differences in olfactory function according to depression (*N* = 108).

Depression	SGDS^1^ Score	*N* (%)	UPSIT^2^	F	*p* ^†^
No	< 8	77 (71.3)	17.92 ± 8.30	0.510	0.477
Yes	≥ 8	31 (28.7)	14.48 ± 8.00		

*^1^SGDS, Short version of the Geriatric Depression Scale; ^2^UPSIT, University of Pennsylvania Smell Identification Test, ^†^ANCOVA analysis results as covariates age, education level, household type, and CDR score.*

### Difference in Olfactory Function According to Cognitive Function

Among the domains of cognitive function, language (*F* = 13.271, *p* < 0.001) showed differences related to olfactory function. Those with cognitive dysfunction in the language domains had significantly lower olfactory function scores than those without cognitive dysfunction. But, there were no significant statistical difference in olfactory function according to attention (*F* = 0.004, *p* = 0.951), visuospatial (*F* = 0.693, *p* = 0.407), memory (*F* = 0.039, *p* = 0.845), and frontal/executive (*F* = 0.387, *p* = 0.535) domain (Table 4).

**TABLE 4:** Differences in olfactory function according to cognitive function (*N* = 108).

SNSB II^1^		*N* (%)	UPSIT	*F*	*p* ^†^
Cognitive domain	Cutoff point				
Attention	< 16	21 (19.4)	13.86 ± 7.30	0.004	0.951
	≥ 16	87 (80.6)	17.68 ± 8.42		
Language	< 16	58 (53.7)	12.90 ± 8.03	13.271	< 0.001
	≥ 16	50 (46.3)	21.62 ± 5.88		
Visuospatial	< 16	49 (45.4)	14.33 ± 8.32	0.693	0.407
	≥ 16	59 (54.6)	19.10 ± 7075		
Memory	< 16	64 (59.3)	15.406 ± 8.901	0.039	0.845
	≥ 16	44 (40.7)	19.159 ± 6.904		
Frontal/executive	< 16	49 (45.4)	14.082 ± 8.946	0.387	0.535
	≥ 16	59 (54.6)	19.305 ± 7.000		

*^†^ANCOVA analysis results as covariates age, education level, household type, and CDR score. ^1^Statistical analysis method.*

### Factors Affecting Olfactory Function

To assess whether the assumptions were satisfied for regression analysis, the linearity between variables was checked using a scatter plot matrix chart, and as a result of examining the autocorrelation of the dependent variable and the multicollinearity between the independent variables, the Durbin-Watson value was 1.938. The tolerances between variables were 0.1 or higher (0.150–0.962), and the variance inflation factor was less than 10 for all variables (1.091–6.666), indicating an absence of multicollinearity between independent variables. The assumptions of both normality and equal variance of residuals were satisfied.

In order to identify the factors that affected olfactory function, which was the dependent variable, a hierarchical multiple regression analysis was performed with independent variables including sex, age, marital status, education level, religion, household type, prescription medication, smoking, depression, and cognitive function. Except for age and education, the other independent variables (sex, marital status, religion, household type, prescription medication, and smoking) were treated as dummy variables in the regression.

In Model 1, the factors that influenced olfactory function in elderly people with dementia were age (β = −0.406, *p* < 0.001), and smoking (yes) (β = −0.205, *p* = 0.025). In Model 2, which included the demographic data as controlled variables with the SGDS and UPSIT, the factors found to influence olfactory function were age (β = −0.265, *p* < 0.001) and the language domain of cognitive function (β = 0.373, *p* < 0.001). The language domain of cognitive function and age had an explanatory power of 39.2% for the olfactory function of elderly people with dementia (*F* = 5.591, *p* < 0.001) (Table 5).

**TABLE 5:** Factors affecting olfactory function.

Variables	Categories	Model 1			Model 2II		
		B	*β*	*t*(*p*)	B	*β*	*t*(*p*)
Sex	Female	0.278	0.017	0.154 (0.878)	1.302	0.079	0.760 (0.449)
Age	-	−0.510	−0.406	−4.317 (< 0.001)	−0.333	−0.265	−2.889 (0.005)
Marital status	Unmarried	2.110	0.126	0.704 (0.483)	1.372	0.082	0.488 (0.627)
Education level	-	1.108	0.162	1.530 (0.129)	1.316	0.192	1.967 (0.052)
Religion	Yes	−1.558	−0.094	−1.067 (0.289)	−1.284	−0.077	−0.955 (0.342)
Household type	Single household	−2.170	−0.121	−0.653 (0.515)	−3.355	−0.187	−1.059 (0.292)
	Spouse and other family	−4.858	−0.236	−1.837 (0.069)	−4.142	−0.201	−1.667 (0.099)
Medication	Yes	2.581	0.071	0.847 (0.399)	−0.195	−0.005	−0.066 (0.947)
Smoking	Yes	−5.859	−0.205	−2.277 (0.025)	−3.955	−0.138	−1.629 (0.107)
SGDS	-				0.120	0.070	0.777 (0.439)
SNSB II ≥ 16	Attention				−0.844	−0.040	−0.464 (0.643)
	Language				6.197	0.373	3.779 (< 0.001)
	Visuospatial				0.861	0.052	0.559 (0.577)
	Memory				0.663	0.039	0.473 (0.637)
	Frontal/executive				1.538	0.092	1.036 (0.303)
		Adj. *R*^2^ = 0.269, *F*(p) = 5.370 (*p* < 0.001)			Adj. *R*^2^ = 0.392, Δ Adj. *R*^2^ = 0.123, *F*(p) = 5.591 (*p* < 0.001)		

## Discussion

This study was conducted to investigate the influence of specific domains of cognitive function and depression on the olfactory function of elderly people with dementia in South Korea in order to facilitate early detection of depression among this population and to use olfaction as an assessment tool to determine the extent of dementia on its associations with specific domains of cognitive function.

In this study, the scores of olfactory function were higher for those living with spouses than for those living with spouses and other family members. However, it is difficult to interpret the finding that olfactory function was directly associated with household type. Participants in this study with mild or moderate CDR scores had lower olfactory function than those with questionable CDR scores. Participants with higher CDR scores may require additional support and thus be more likely to live with additional family members. Because, Korean adult children traditionally have the responsibility of caring for their elderly parents, especially their ill parents (Hong and Kim, 2008). Therefore, it might have been the case that the participants in this study lived with other family members because they had difficulties in their daily lives.

In the results of this study, even though education level was not a factor influencing olfactory function, the olfactory function scores in those who had college-level education were statistically significantly higher than in those who were unschooled. A scoping review on associations between olfactory function and sociodemographic characteristics reported that the association between education level and olfactory function was unclear, since the results of many studies were not consistent (James et al., 2021). In a study that analyzed UPSIT scores in a normal population in Brazil, there was no significant association between education level and olfactory function, but it was reported that the participants’ economic status was associated with their ability to distinguish specific odors (Silveira-Moriyama et al., 2010). Since the UPSIT involves identifying various types of odors, the results may vary depending on individual’s education level or the experience of exposure to a specific odor (Silveira-Moriyama et al., 2010; Fornazieri et al., 2019; Park et al., 2021). Therefore, identifying culturally unfamiliar test odors and modifying and simplifying the test so that anyone can easily distinguish the options will be helpful to obtain more accurate test results.

In the current study, olfactory function scores were significantly lower when the CDR test scores were mild or moderate than when the scores were questionable. Impaired olfactory function can lead to decreased appetite and imbalanced nutritional intake (Lee et al., 2007), difficulty identifying dangerous situations due to the inability to recognize odors such as spoiled food or smoke, and low quality of life and daily living behaviors (Seo et al., 2009; Yuan and Slotnick, 2014). For elderly people with dementia, the ability to perform daily living activities gradually decreases due to cognitive decline, thus compromising their quality of life (Arvanitakis et al., 2019). Since these problems can be exacerbated by olfactory function impairment, olfactory dysfunction or decline should not be overlooked. Therefore, it may prove useful to assess the integrity of olfactory function in elderly people with dementia at an early stage and to develop effective strategies to educate people and address physical and psychological problems that may result from decreased olfactory function.

The mean score for olfactory function was 14.48 points for participants with depression and 17.92 points for participants without depression. Both groups had low olfactory function scores, but the difference was not statistically significant. Participants in this study were elderly people with dementia. Since olfactory function decreases with cognitive dysfunction among this population (Marin et al., 2018), it can be understood that both groups had low olfactory function scores regardless of the presence or absence of depression. In a systematic review of the literature on the relationship between depression and olfactory function, only 60% of studies included in the analysis found that overall olfactory function scores were significantly lower for participants with depression than for participants without depression, showing inconsistent results from study to study (Taalman et al., 2017). In addition, in a study that examined olfactory function in patients diagnosed with depressive disorder, it was found that a longer period of depression and recurrence of depression were related to decreased olfactory function, suggesting a correlation between the severity of depression and olfactory dysfunction (Pabel et al., 2018). Therefore, it is necessary to conduct further research that appropriately reflects the duration of depression or its recurrence.

Although, smoking was not a factor influencing olfactory function in Model 2, there was a significant effect of smoking on olfactory function in Model 1. Smokers had higher olfactory function than non-smokers. However, a previous systematic review indicated that current smoking is associated with increased risk of olfactory dysfunction in general population (Ajmani et al., 2017). In contrast to former studies, we did not find the increased risk of olfactory dysfunction due to smoking status. Our differing findings may be induced by the small sample size of smokers and the relative imbalance in smoking status in participants. Therefore, further study is needed in larger populations to identify the relationship between smoking and olfactory function in dementia patients.

Furthermore, the age of elderly people with dementia was found to be an influencing factor for decreased olfactory function. Age is an important factor influencing olfactory function. Although decreased olfactory function is an early symptom of dementia (Marin et al., 2018), the likelihood of developing olfactory dysfunction was found to increase steadily with age in elderly people regardless of the presence or absence of dementia (Palmquist et al., 2020). Therefore, it is difficult to determine olfactory dysfunction based only on olfactory function test scores (Devanand, 2016), and we believe that, in order to identify significant declines in olfactory function in elderly people with dementia, an age-adjusted scoring standard should be developed.

The analysis of the five domains of cognitive function showed that only the language domain was a factor influencing the olfactory function of elderly people with dementia. Several studies have reported that language ability was associated with olfactory dysfunction (Larsson et al., 2005; Tonacci and Billeci, 2018; Turana et al., 2020). On the other hand, a cross-sectional study has shown that verbal intellectual abilities like analogies and synonyms was unrelated to olfactory function (Larsson et al., 2000). Olfactory information from olfactory sensory neurons passes through the entorhinal cortex to the frontal lobes to process information. Through this process, odors can be recognized and distinguished (Lee et al., 2007). A recent study reported that the olfactory system and lexical brain system are strongly linked to each other in the process of recognizing, distinguishing, and naming odors (Zald and Pardo, 2000). High verbal skill is required to perform the odor identification test (Frasnelli et al., 2010) and language fluency might be related to odor identification (Devanand et al., 2015). Since elderly people with dementia may have limited language skills, developing a non-verbal test to identify olfactory function may be necessary.

In this study, the other cognitive domains, including attention, memory, visuospatial, and frontal/executive, were not related to olfactory function. Given that damage to the frontostriatal circuit of the brain causes problems for executive and olfactory function (Parrao et al., 2012), the executive domain has been associated with olfactory function in previous research (Hedner et al., 2010). The memory domain (Hedner et al., 2010; Palmquist et al., 2020) has also been associated with olfactory function, as the hippocampus, which is used in distinguishing and discriminating scents, is also a brain structure that processes memory (Lee, 2017). The participants in the current study were patients with various types of dementia. Considering that the functioning of each cognitive domain differs according to the cause of dementia (Seo, 2018), there were limitations in identifying the relationship between each domain of cognitive function and olfactory function. Therefore, it is necessary in the future for studies to identify the causative diseases of dementia and to reflect such causes when examining the relationship between each domain of cognitive function and olfactory function.

The limitation of this study is the lack of comparison between those with dementia and the normal population. A comparative analysis of normal aging individuals and those with varying degrees of dementia by itself or associated with AD and or PD would greatly improve this study. Moreover, even though the UPSIT used in this study is an internationally common olfactory function test tool that has been published in 30 languages, it is difficult to exclude the possible issue of unfamiliarity. In a previous Korean study reported that odor identification rate could be low regardless of the clinical characteristics of patients due to cultural differences and individual scent familiarity (Kim, 2019). As increasing evidence emerges for an association between cognitive dysfunction and olfactory dysfunction, the development and implementation of an olfactory function test that is culture-friendly for older people with cognitive dysfunction may be urgent.

The results of this study could support the suggestion that accurate assessment of olfaction could aid in precision in diagnosis of dementia. Furthermore, this study confirmed the relationship between the language domain of cognitive function and olfactory dysfunction. The range of olfactory dysfunction might be influenced by specific cognitive functions.

## Conclusion

In conclusion, we could not address the relationship between depression and olfactory function, but our results support the hypothesis that language cognitive function decline and old age accompany the occurrence of olfactory dysfunction in elderly patient with dementia. Our finding suggested that olfactory function test considering cultural factor and verbal ability should be developed. Also, further studies are need to identify the association with characteristics of depression and olfactory function with the demented elderly.

## Author’s Note

The data of this study were presented in a previous study by Hyangjeong Park, Heejeong Kim, Sisook Kim, and Hyegyeong Cha (2021), entitled “The Association between Olfactory Function and Cognitive Impairment in Older Persons with Cognitive Impairments: A Cross Sectional Study”. Healthcare, 9(4), 399; https://doi.org/10.3390/healthcare9040399. However, in the current study, we conducted additional analyses of the participants’ Seoul Neuropsychological Screening Battery (SNSB II) test data and the short version of the Geriatric Depression Scale (SGDS) data, which were provided in cooperation with the hospital, to confirm the relationship between olfaction, depression, and specific domains of cognitive function (attention, language, visuospatial, memory, and frontal/executive). In this respect, this study can be differentiated from the previous one, which only investigated the relationship between cognitive decline and olfactory function, and the present study can be considered a more thorough development of the previous work.

## Data Availability Statement

The original contributions presented in the study are included in the article/supplementary material, further inquiries can be directed to the corresponding author/s.

## Ethics Statement

The studies involving human participants were reviewed and approved by the institutional review board of the Namseoul University located in Cheonan (IRB No: 1041479-HR-20190-002 on 25 March 2019). The studies involving human participants were carried out in accordance with the Declaration of Helsinki. The patients/participants provided their written informed consent to participate in this study.

## Author Contributions

HC contributed to the conception and design of the study and organized the database. SK performed the statistical analysis. HC, SK, and YS wrote the first draft of the manuscript and wrote sections of the manuscript. All authors contributed to manuscript revision, read, and approved the submitted version.

## Conflict of Interest

The authors declare that the research was conducted in the absence of any commercial or financial relationships that could be construed as a potential conflict of interest.

## Publisher’s Note

All claims expressed in this article are solely those of the authors and do not necessarily represent those of their affiliated organizations, or those of the publisher, the editors and the reviewers. Any product that may be evaluated in this article, or claim that may be made by its manufacturer, is not guaranteed or endorsed by the publisher.
